# A unified CPG-based and multi-agent control framework for low-cost quadruped robots

**DOI:** 10.1371/journal.pone.0356505

**Published:** 2026-08-25

**Authors:** Likai Wu, Meina Zhang, Wenzhe Wang

**Affiliations:** 1 School of Electrical Engineering, Shandong Huayu University of Technology, Dezhou, Shandong, China; 2 Department of Naval Architecture and Ocean Engineering, Pusan National University, Busan, South Korea; Qingdao University, CHINA

## Abstract

Quadruped robots have gained significant attention due to their superior mobility on uneven and unstructured terrains, offering potential applications in inspection, search and rescue, and field exploration. However, achieving robust locomotion on low-cost platforms remains challenging because of constraints in stability, adaptability, sensing quality, and onboard computation. In this work, we present an integrated motion-control framework that combines biologically inspired Central Pattern Generators (CPGs), a multi-agent reinforcement learning coordination layer, and low-cost hardware adaptation to enable reliable and efficient quadruped locomotion. The proposed framework uses CPGs as structured gait priors for rhythmic leg motion, models each leg as a coordinated agent with a shared-parameter residual policy, and incorporates actuator abstraction and safety-aware command projection. The low-cost merit specifically concerns online deployment: the four legs share a single 39,560-parameter actor (approximately 155 KiB in 32-bit precision), evaluated at 50 Hz from compact proprioceptive observations, while the centralized critic, simulation infrastructure, external motion capture, vision-based terrain perception, direct torque sensing, and online dynamics optimization are not required on the robot. We validate the approach in both simulation and on a physical low-cost quadruped robot across obstacles, ramps, stairs, and uneven terrain. Experimental results demonstrate that the integrated system improves locomotion stability, energy efficiency, and terrain adaptability compared with baseline controllers, highlighting the effectiveness of combining a structured gait prior, lightweight residual coordination, and hardware-aware deployment for practical quadruped locomotion.

## 1 Introduction

Quadruped robots have emerged as one of the most promising classes of legged platforms for operation in uneven, discontinuous, and unstructured environments. Compared with wheeled or tracked robots, quadrupeds can exploit discrete foothold selection, body posture regulation, and gait reconfiguration to traverse obstacles, slopes, stairs, and compliant terrain, making them attractive for infrastructure inspection, search and rescue, industrial monitoring, and field exploration tasks [1–6]. Over the past decade, advances in mechanical design, actuation, state estimation, perception, and control have enabled quadrupeds to progress from laboratory demonstrators to highly agile systems capable of dynamic trotting, jumping, climbing, and autonomous terrain negotiation [7–14].

Despite this rapid progress, achieving robust locomotion on *low-cost* quadruped platforms remains a challenging problem. Many state-of-the-art results have been demonstrated on platforms equipped with high-performance actuators, accurate proprioceptive and exteroceptive sensing pipelines, and substantial onboard or offline computational resources [9–13,15–17]. In contrast, low-cost platforms usually face tighter constraints in actuator bandwidth, sensing reliability, embedded computation, and mechanical consistency, which can significantly degrade gait stability, disturbance rejection, and terrain adaptability. Open and affordable systems such as Stanford Doggo, Stanford Pupper, OpenQuadruped, and recent low-cost 3D-printed quadrupeds have greatly improved accessibility for research and education, yet the gap between accessibility and robust field-capable locomotion remains substantial [18–22].

Among the many locomotion paradigms proposed for legged systems, central pattern generators (CPGs) remain particularly attractive because they provide a compact and biologically grounded mechanism for generating rhythmic motion. Inspired by neural oscillators found in animal locomotion, CPG-based methods can produce coordinated limb trajectories with relatively low computational cost and strong interpretability [23–27]. This makes them especially suitable for hardware-constrained platforms where lightweight control architectures are desirable. Prior studies have shown that CPG-based control can support adaptive walking, reflex integration, layered rhythm generation, and spontaneous gait transitions in quadruped robots [28–31]. However, pure CPG approaches may still struggle when facing strongly varying terrain, external disturbances, and the practical uncertainties introduced by inexpensive sensors and actuators.

In parallel with biologically inspired control, model-based and learning-based locomotion methods have achieved impressive dynamic performance. Optimization-based frameworks such as convex model predictive control, online nonlinear motion optimization, and real-time motion planning have improved tracking accuracy, dynamic balance, and disturbance rejection in challenging terrain [10,15,32,33]. Learning-based approaches have further expanded the capability envelope of quadrupeds by enabling agile motor skills, perceptive locomotion, deformable-terrain traversal, and highly dynamic navigation [11–14,17,34,35]. Nevertheless, these methods often rely on large-scale training, carefully tuned simulation-to-real transfer, high-rate computation, or sophisticated sensing stacks, which may not be readily available on low-cost robots.

For low-cost quadrupeds, another critical issue is state estimation under noisy sensing and uncertain contact conditions. Reliable locomotion on uneven terrain requires sufficiently accurate estimates of body pose, velocity, and contact state, yet inexpensive platforms often suffer from sensor drift, actuator backlash, and reduced sensing redundancy. Existing research has demonstrated that heterogeneous sensor fusion, contact-aided invariant filtering, and modular multi-sensor estimators can substantially improve state estimation for legged systems [36–39]. In addition, perception-less or weakly perceptive terrain-adaptive strategies suggest that robust locomotion does not always require a heavyweight perception pipeline, provided that the control architecture can exploit structured dynamics, feedback integration, and suitable abstractions of the actuation layer [16,40].

A further issue concerns the *coordination architecture* of locomotion control. While many quadruped controllers are formulated in a centralized manner, stable global locomotion can also emerge from coordinated local interactions among leg subsystems. In quadruped locomotion, this view appears in layered CPG structures, reflex-mediated adaptation, self-organized gait transitions, and more recent formulations that combine oscillatory priors with decentralized coordination concepts [29–31,41,42]. Motivated by this perspective, we formulate the present controller as a leg-level multi-agent system: each leg is treated as an agent with its own local state, local residual action, and shared coordination objective, while a shared policy architecture and global state bus maintain coherence across the four agents. Such a formulation is particularly appealing for low-cost quadrupeds because it preserves modularity, improves tolerance to sensing or actuation variation across legs, and supports hardware abstraction without redesigning the full locomotion stack.

Therefore, an important gap remains between three lines of research that are often studied separately: biologically inspired rhythmic control, distributed or modular coordination, and practical low-cost hardware adaptation. Existing CPG-based studies often emphasize gait generation and rhythmic stability, but less frequently address deployability on constrained hardware in diverse terrain. Conversely, high-performance model-based and learning-based systems demonstrate remarkable agility, but commonly assume premium hardware and richer sensing/computational resources. Open low-cost quadruped platforms improve accessibility, yet many are primarily intended as research or educational platforms rather than as unified solutions for terrain-adaptive locomotion [6,19–21,27,35].

To address this gap, this paper proposes a CPG-conditioned leg-level residual coordination framework for low-cost quadruped locomotion. The central idea is not to use the CPG, reinforcement learning, and CTDE as three independent modules, but to make the CPG oscillator state serve as a structured coordination prior for the residual multi-agent policy. In the proposed controller, the CPG provides not only nominal joint trajectories, but also phase, amplitude, contact schedule, step-frequency, and swing-height information that are explicitly exposed to the leg agents. Each leg agent then learns bounded residual corrections to its local joint targets and bounded proposals for gait-parameter adaptation. These leg-level proposals are aggregated into common frequency and swing-height updates, so that local adaptation can occur without destroying the global gait rhythm.

This design differs from conventional CPG controllers that rely mainly on fixed oscillator parameters or reflex feedback, and also differs from monolithic residual RL controllers that directly learn full-body corrections on top of a nominal gait. The proposed method introduces a closed-loop coupling between the rhythmic prior and the residual multi-agent policy: the CPG constrains the learning problem to a stable gait manifold, while the leg-level residual agents adapt the CPG-generated motion online according to contact, body-attitude, and local joint feedback. This coupling is further constrained by actuator limits, command-rate limits, and safety projection, making the method suitable for low-cost servo-based quadruped platforms with limited sensing and computation.

In this paper, the term *low-cost* refers specifically to the online sensing, computation, and hardware-integration burden rather than to the expense of offline simulation training. The core advantages of the algorithm are fourfold. First, the CPG generates the nominal rhythmic motion, so the learned component only predicts bounded four-dimensional residual actions for each leg instead of generating the complete gait. Second, all four legs reuse one shared actor, avoiding four independently stored policies. Third, CTDE moves the centralized critic and the expensive training infrastructure offline; only the compact actor is retained during execution. Fourth, the online controller uses low-dimensional proprioceptive feedback and fixed-complexity operations—CPG integration, planar inverse kinematics, residual addition, clipping, rate limiting, and filtering—without external motion capture, direct torque sensing, vision-based terrain reconstruction, or iterative whole-body optimization. These design choices constitute the algorithmic basis of the low-cost claim.

The main contributions of this work are as follows:

1. We propose a CPG-conditioned residual multi-agent control mechanism in which the oscillator phase, amplitude, contact schedule, and gait parameters are directly used to condition leg-level residual policies. This allows the learned policy to adapt the CPG gait online rather than replacing it with a fully learned controller.
2. We design a leg-level coordination strategy in which each agent outputs both local joint residuals and gait-parameter adaptation proposals. The frequency and swing-height proposals from the four legs are aggregated into common gait updates, preserving whole-body rhythmic coherence while allowing local terrain responses.
3. We formulate a low-complexity CTDE implementation with a 23-dimensional local observation, a four-dimensional residual action, and one 39,560-parameter actor shared by all legs. The shared actor requires approximately 155 KiB in 32-bit precision and approximately $7.83\times 10^{6}$ multiply–accumulate operations per second when evaluated for four legs at 50 Hz; the centralized critic is discarded after training.
4. We implement the controller using proprioceptive sensing, closed-form CPG and inverse-kinematics updates, and hardware-aware command projection on a low-cost servo-based quadruped. The online method does not require external motion capture, direct torque sensors, vision-based terrain perception, or iterative model-predictive optimization, and is evaluated over obstacles, ramps, stairs, and uneven terrain.

## 2 Methodology

In this work, we propose an integrated motion-control architecture for quadruped robots that unifies three complementary aspects: CPG-based locomotion generation, explicit multi-agent coordination, and deployment on low-cost hardware platforms.

### 2.1 System overview

**Fig 1:**
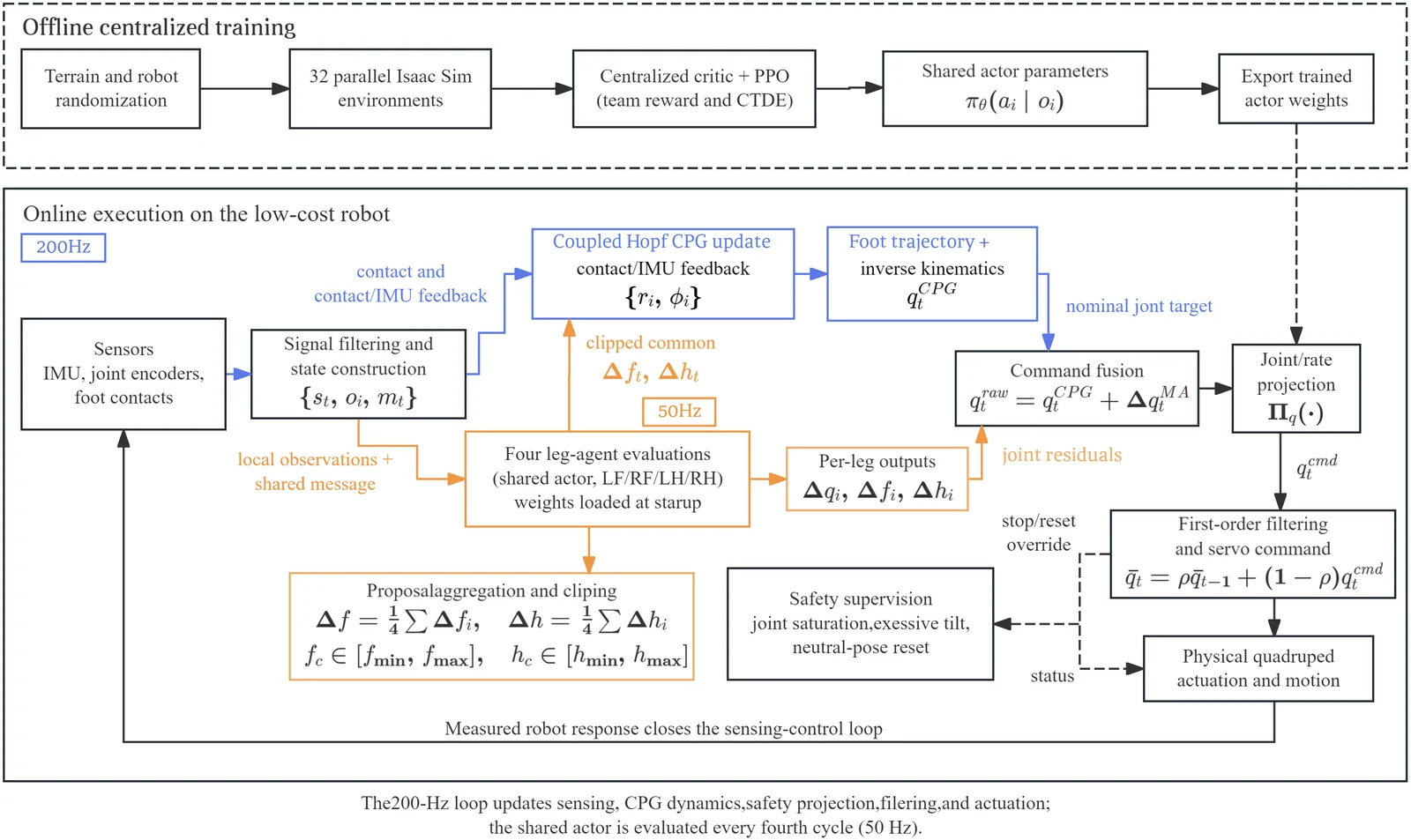
Detailed implementation pipeline of the proposed CPG-conditioned leg-level multi-agent controller. The upper panel shows offline centralized training in Isaac Sim, during which the shared actor and centralized critic are optimized using PPO. After training, only the shared actor parameters are deployed on the physical robot. The lower panel shows the online two-rate control architecture. Sensor acquisition, state construction, CPG integration, inverse kinematics, safety projection, command filtering, and servo actuation operate at 200 Hz, whereas the shared actor is evaluated independently for the four leg agents at 50 Hz. The leg-level frequency and swing-height proposals are aggregated and clipped before being fed back to the CPG. The nominal and residual joint targets are then fused, projected to hardware-safe limits, filtered, and transmitted to the servo controller, thereby closing the sensing–control loop.

The proposed implementation consists of two stages: offline centralized training and online two-rate execution, as illustrated in Fig 1. During offline training, the shared leg-level actor and centralized critic are optimized in 32 parallel Isaac Sim environments under the centralized-training–decentralized-execution paradigm. Terrain properties, robot parameters, and external disturbances are randomized during training. After convergence, only the trained shared actor parameters are exported to the embedded controller; the centralized critic is not required during online execution.

During online execution, proprioceptive measurements are acquired and synchronized at 200 Hz. The IMU orientation and angular velocity, joint states, and foot-contact signals are filtered to construct the global state $s_{t}$, the four local observations $\{o_{i}{\}}_{i=1}^{4}$, and the shared coordination message $m_{t}$. The low-level branch integrates the four coupled Hopf oscillators using contact and body-attitude feedback, maps the oscillator states to desired Cartesian foot trajectories, and applies inverse kinematics to obtain the nominal joint target ${𝐪}_{t}^{CPG}$.

The high-level multi-agent policy is evaluated every fourth low-level cycle, corresponding to 50 Hz. At each policy update, the same shared actor is evaluated once for each of the LF, RF, LH, and RH leg agents. Each agent outputs two local joint residuals and two gait-adaptation proposals, $\Delta f_{i}$ and $\Delta h_{i}$, for the step frequency and swing height. The four frequency and swing-height proposals are averaged and clipped to hardware-safe intervals before being fed back to the CPG. The latest residual actions and common gait parameters are held constant between two consecutive policy updates, while the CPG and servo loops continue to operate at 200 Hz.

At every low-level cycle, the nominal joint target and the most recently available multi-agent residual command are combined as


$$\begin{array}{c} {𝐪}_{t}^{cmd}=\Pi_{𝒬}\;\left({𝐪}_{t}^{CPG}+\Delta {𝐪}_{t}^{MA}\right), \end{array}$$ (1)

where ${𝐪}_{t}^{CPG}\in {\mathbb{R}}^{8}$ is the nominal joint target generated by the CPG and inverse-kinematics modules, $\Delta {𝐪}_{t}^{MA}\in {\mathbb{R}}^{8}$ is the concatenated residual command generated by the four leg agents, and $\Pi_{𝒬}(\cdot )$ applies joint-position and command-rate limits before the command is passed to the hardware interface. A first-order filter is subsequently applied to suppress servo-command chattering.

### 2.2 CPG-based gait generation module

Each leg is associated with one nonlinear oscillator with amplitude $r_{i}$ and phase $\phi_{i}$, where i∈{LF,RF,LH,RH}. We use a Hopf-type formulation:


$$\begin{array}{c} {\dot{r}}_{i}=\alpha_{r}\left(\mu_{i}-r_{i}^{2}\right)r_{i}+k_{r}\left(c_{i}-{\bar{c}}_{i}\right), \end{array}$$ (2)

$$\begin{array}{c} {\dot{\phi }}_{i}=2\pi f_{i}+\sum_{j\neq i}w_{ij}\sin\;\left(\phi_{j}-\phi_{i}-\Phi_{ij}\right)+k_{\phi }\;\delta_{i}, \end{array}$$ (3)

where $\alpha_{r}$ is the amplitude convergence gain, $\mu_{i}$ is the nominal oscillation radius, $f_{i}$ is the step frequency, $w_{ij}$ is the coupling weight between oscillators *i* and *j*, $\Phi_{ij}$ is the desired phase difference, $c_{i}\in \{0,1\}$ is the measured foot-contact state, and ${\bar{c}}_{i}$ is the desired contact state inferred from the current gait phase. The feedback term


$$\begin{array}{c} \delta_{i}=\lambda_{c}\left(c_{i}-{\bar{c}}_{i}\right)+\lambda_{p}\theta_{pitch}+\lambda_{r}\theta_{roll} \end{array}$$ (4)

uses foot contact and IMU pitch/roll to slightly advance or delay the oscillator when terrain or body attitude deviates from the nominal gait. The desired contact signal is computed from the oscillator phase as


$$\begin{array}{c} {\bar{c}}_{i}=\left\{ \begin{array}{cc} 1, & \sin\phi_{i}\leq 0,\\ 0, & \sin\phi_{i}>0, \end{array}\right. \end{array}$$ (5)

where ${\bar{c}}_{i}=1$ denotes the nominal stance part of the gait cycle. This definition makes the contact feedback term in Eq. (4) interpretable as a phase correction driven by the mismatch between expected and measured contact.

For the trot gait used in the experiments, the desired phase vector is


$$\begin{array}{c} \phi^{⋆}=[0,\;\pi ,\;\pi ,\;0], \end{array}$$ (6)

for the ordered leg set [LF,RF,LH,RH], so that diagonal legs move in phase. The phase-offset matrix is defined by $\Phi_{ij}=\phi_{j}^{⋆}-\phi_{i}^{⋆}$, and in the implementation we use symmetric all-to-all coupling with $w_{ij}=1$ for i≠j and $w_{ii}=0$. The nominal oscillator parameters used in our experiments are $\alpha_{r}=20$, $\mu_{i}=1$, and base step frequency *f*_0_ = 1.6 Hz.

The oscillator states are mapped to Cartesian foot trajectories as


$$\begin{array}{c} x_{i}^{d}=x_{0,i}+A_{x}r_{i}\cos\phi_{i},\;z_{i}^{d}=z_{0,i}+h_{t}\max\left(0,\sin\phi_{i}\right), \end{array}$$ (7)

with $y_{i}^{d}=y_{0,i}$ fixed by the robot geometry. Here $A_{x}$ is the stride amplitude and $h_{t}$ is the swing-foot clearance. In the nominal setting, $A_{x}=0.035$ m and *h*_0_ = 0.025 m. The desired foot positions are converted to hip and knee targets by planar inverse kinematics for each leg, giving ${𝐪}_{t}^{CPG}$ in Eq. (1).

### 2.3 CPG parameter tuning and terrain-adaptive adjustment

The CPG parameters were selected before reinforcement learning using a three-stage tuning procedure. First, oscillator-internal parameters ($\alpha_{r}$, $\mu_{i}$, $w_{ij}$, and $\Phi_{ij}$) were chosen to ensure fast convergence to the desired trot limit cycle and stable diagonal-leg phase locking. Second, kinematic parameters ($A_{x}$, *h*_0_, and *f*_0_) were swept on flat ground to obtain a nominal gait that matched the target speed without joint saturation. Third, the feedback gains ($k_{r}$, $k_{\phi }$, $\lambda_{c}$, $\lambda_{p}$, and $\lambda_{r}$) were increased gradually under slope, obstacle, and push disturbances, and the smallest gains that reduced contact mismatch and body-attitude drift without introducing command oscillation were retained. The final parameter values used in the experiments are summarized in Table 1.

**Table 1:** Controller parameters used in the experiments.

Parameter	Value	Role
$\alpha_{r}$	20	Hopf amplitude convergence gain.
$\mu_{i}$	1.0	Nominal oscillator radius.
$w_{ij}$	1 for i≠j, 0 for *i* = *j*	Inter-oscillator phase coupling.
$\phi^{⋆}$	[0,π,π,0]	Desired trot phase vector.
*f*_0_	1.6 Hz	Nominal step frequency.
$f_{\min},f_{\max}$	1.0, 2.2 Hz	Frequency clipping bounds.
$A_{x}$	0.035 m	Stride amplitude.
*h*_0_	0.025 m	Nominal swing clearance.
$h_{\min},h_{\max}$	0.015, 0.045 m	Swing-height clipping bounds.
$k_{r}$	0.10	Contact feedback gain for oscillator amplitude.
$k_{\phi }$	0.20	Feedback gain for oscillator phase correction.
$\lambda_{c}$	0.50	Contact mismatch weight in the phase-feedback term.
$\lambda_{p},\lambda_{r}$	0.08, 0.08	Pitch and roll feedback weights in the phase-feedback term.
ρ	0.8	Command-filter coefficient.
$\eta_{f},\eta_{h}$	0.05, 0.05	Coordination-variance weights for frequency and swing-height proposals.

Terrain adaptation is implemented continuously rather than by manually switching parameter sets for different terrains. Contact and IMU deviations perturb the oscillator through Eqs. (2)–(4), while the multi-agent residual policy adjusts the common step frequency and swing height through $\Delta f_{i}$ and $\Delta h_{i}$. The resulting frequency and clearance are clipped to hardware-safe intervals in Eq. (14). Thus, ramps primarily induce phase and frequency corrections through body pitch and contact timing, whereas obstacles and stairs mainly increase the learned swing-height correction when premature contact or missed clearance is detected.

A one-at-a-time sensitivity check was used to verify that the controller was not dependent on a single narrow parameter setting. The most sensitive parameters were *f*_0_, $A_{x}$, and *h*_0_, because they directly determine forward speed, reachable footholds, and obstacle clearance. Moderate changes in $\alpha_{r}$ and $w_{ij}$ preserved the trot pattern as long as the oscillator remained phase locked. Feedback gains above the selected values improved recovery in some disturbed trials but increased command chattering on the physical robot. For this reason, conservative gains were used and only the residual policy was allowed to make bounded online adjustments.

### 2.4 Leg-level multi-agent coordination architecture

We explicitly model the four legs as four cooperating agents,


$$\begin{array}{c} 𝒜=\{a_{LF},a_{RF},a_{LH},a_{RH}\}. \end{array}$$ (8)

Each agent controls one leg and outputs residual corrections for the hip and knee joints of that leg. To balance modularity and coordination, we use centralized training with decentralized execution and shared actor parameters across all agents. During training, each agent has access to a global state for value estimation, whereas at execution time each agent acts from its local observation augmented with a compact shared coordination signal.

For leg agent *i*, the local observation is defined as


$$\begin{array}{c} o_{i}=[q_{i},\;{\dot{q}}_{i},\;r_{i},\;\phi_{i},\;c_{i},\;{\bar{c}}_{i},\;\theta_{roll},\;\theta_{pitch},\;\omega_{x},\;\omega_{y},\;a_{i,t-1},\;v_{x}^{⋆},\;m_{t}], \end{array}$$ (9)

where $q_{i}\in {\mathbb{R}}^{2}$ and ${\dot{q}}_{i}\in {\mathbb{R}}^{2}$ are the hip and knee states of leg *i*, $r_{i}$ and $\phi_{i}$ are the corresponding oscillator states, $c_{i}$ is the measured contact state, ${\bar{c}}_{i}$ is the desired contact state from the gait generator, $\left(\theta_{roll},\theta_{pitch}\right)$ and $\left(\omega_{x},\omega_{y}\right)$ are body-attitude measurements from the IMU, $a_{i,t-1}$ is the previous action of the same agent, $v_{x}^{⋆}$ is the target forward speed, and $m_{t}$ is a shared coordination message broadcast through the global state bus.

The shared coordination message is


$$\begin{array}{c} m_{t}=[{\bar{f}}_{t},\;{\bar{h}}_{t},\;{\bar{c}}_{LF},\;{\bar{c}}_{RF},\;{\bar{c}}_{LH},\;{\bar{c}}_{RH}], \end{array}$$ (10)

where ${\bar{f}}_{t}$ is the common step-frequency command, ${\bar{h}}_{t}$ is the common swing-height command, and ${\bar{c}}_{i}$ indicates the desired contact schedule of each leg. This message allows each agent to remain aware of the global gait pattern without requiring a fully centralized action output.

Each agent outputs a local residual action


$$\begin{array}{c} a_{i,t}=[\Delta q_{i,1},\;\Delta q_{i,2},\;\Delta f_{i},\;\Delta h_{i}], \end{array}$$ (11)

where $\Delta q_{i,1}$ and $\Delta q_{i,2}$ are residual corrections for the hip and knee joints of leg *i*, $\Delta f_{i}$ is a local contribution to step-frequency adaptation, and $\Delta h_{i}$ is a local contribution to swing-height adaptation. The joint residual vector applied to the full robot is then


$$\begin{array}{c} \Delta {𝐪}_{t}^{MA}=[\Delta q_{LF,1},\Delta q_{LF,2},\Delta q_{RF,1},\Delta q_{RF,2},\Delta q_{LH,1},\Delta q_{LH,2},\Delta q_{RH,1},\Delta q_{RH,2}{]}^{⊤}. \end{array}$$ (12)

The common gait parameters are obtained by averaging the leg-level proposals,


$$\begin{array}{c} \Delta f_{t}=\frac{1}{4}\sum_{i=1}^{4}\Delta f_{i},\;\Delta h_{t}=\frac{1}{4}\sum_{i=1}^{4}\Delta h_{i}, \end{array}$$ (13)

so that


$$\begin{array}{c} f_{t}=clip(f_{0}+\Delta f_{t},f_{\min},f_{\max}),\;h_{t}=clip(h_{0}+\Delta h_{t},h_{\min},h_{\max}). \end{array}$$ (14)

This aggregation preserves a coherent whole-body gait while allowing each leg to contribute to adaptation. In the implementation, joint residuals are bounded by $|\Delta q_{i,k}|\leq 0.25$ rad, frequency proposals by $|\Delta f_{i}|\leq 0.4$ Hz, and swing-height proposals by $|\Delta h_{i}|\leq 0.02$ m before the final safety projection in Eq. (1).

The local observation in Eq. (9) contains leg-specific variables ($q_{i}$, ${\dot{q}}_{i}$, $r_{i}$, $\phi_{i}$, $c_{i}$, and ${\bar{c}}_{i}$), low-dimensional body-stability variables ($\theta_{roll}$, $\theta_{pitch}$, $\omega_{x}$, and $\omega_{y}$), the previous local action, the commanded speed, and the compact coordination message $m_{t}$. This design intentionally avoids giving each decentralized actor the full 8-joint robot state at runtime, which would increase redundancy and make the policy more sensitive to missing or noisy channels. At the same time, it does not omit the global information needed for gait coherence: the shared message carries the common phase schedule, frequency, and swing-height commands, while the IMU terms provide whole-body balance information. The action space is residual rather than absolute because the CPG already provides a dynamically consistent nominal gait; the policy therefore learns bounded corrections for local joint targets and bounded proposals for global gait parameters. During training, the centralized critic receives the global state in Eq. (15), which contains the full robot posture, contacts, oscillator states, and previous actions. This CTDE split reduces partial-observability problems during learning while retaining decentralized execution on the embedded hardware. The main missing information is exteroceptive terrain geometry; the present controller therefore targets proprioceptive terrain adaptation and not vision-guided foothold planning.

### 2.5 Multi-agent network architecture and training

The shared actor network $\pi_{\theta }(a_{i}|o_{i})$ is implemented as a multilayer perceptron with two hidden layers of 256 and 128 units and ReLU activations. The same actor parameters are shared across the four leg agents. A centralized critic $V_{\psi }(s_{t})$ is trained from the global state


$$\begin{array}{c} s_{t}=[{𝐪}_{t},{\dot{𝐪}}_{t},\theta_{t}^{rp},\omega_{t}^{b},{𝐜}_{t},{𝐫}_{t},\phi_{t},{𝐚}_{t-1},v_{x}^{⋆}], \end{array}$$ (15)

which is the same global observation used for full-system value estimation. We therefore adopt a centralized-training/decentralized-execution paradigm: the critic evaluates whole-body coordination during training, while each leg executes from its own local observation and shared message at runtime.

The actor outputs the mean of a Gaussian policy over the 4-dimensional action of each leg agent together with a learned log-standard-deviation vector. The critic outputs a scalar state value. Optimization is performed using a PPO-style clipped objective with clip ratio 0.2, discount factor γ=0.99, and generalized-advantage parameter λ=0.95. The policy was optimized with Adam using an initial learning rate of $3\times 10^{-4}$, linear learning-rate decay, rollout length of 2048 policy steps per update, minibatch size of 4096 samples, 10 optimization epochs per update, entropy coefficient 0.01, value-loss coefficient 0.5, and gradient-norm clipping at 0.5. Training used 32 parallel simulated environments and was stopped after $3.0\times 10^{7}$ simulator steps or when the evaluation success rate changed by less than 2% over ten consecutive evaluation windows. These implementation settings are summarized here to make the learning procedure reproducible.

**Deployment complexity.** The local observation in Eq. (9) has 23 scalar elements and the action in Eq. (11) has four elements. For the 23–256–128–4 actor, the total number of trainable parameters is


$$\begin{array}{c} N_{actor}=(23\times 256+256)+(256\times 128+128)+(128\times 4+4)+4=39,560, \end{array}$$ (16)

where the last four parameters are the learned log-standard deviations. This corresponds to approximately 155 KiB when stored in 32-bit floating-point format. One deterministic actor evaluation requires 39,168 multiply–accumulate operations in the three fully connected layers. Evaluating the shared actor for four legs at 50 Hz therefore requires approximately $7.83\times 10^{6}$ multiply–accumulate operations per second, excluding the comparatively small fixed-cost CPG and inverse-kinematics operations. Sharing one actor across four legs reduces actor storage by 75% relative to deploying four independent actors of the same architecture. The centralized critic, PPO optimizer, rollout buffers, and simulation environments are used only during training and are not deployed on the physical robot. These counts quantify model size and arithmetic complexity; processor-specific latency and electrical power depend on the embedded software and hardware implementation.

To encourage both local leg effectiveness and global locomotion quality, we use a shared team reward supplemented with a coordination regularizer. The total reward is


$$\begin{array}{c} r_{t}=1.5r_{vel}+0.8r_{post}+0.3r_{cont}+0.2r_{clr}-0.002r_{pow}-0.1r_{slip}-0.05r_{sm}-0.1r_{coord}+r_{term}, \end{array}$$ (17)

where each term is computed at the control step as follows:


$$\begin{array}{c} \begin{array}{ccc} r_{vel} & = & \exp\left[-\frac{(v_{x}-v_{x}^{⋆}{)}^{2}}{\sigma_{v}^{2}}\right], \end{array} \end{array}$$ (18)

$$\begin{array}{c} \begin{array}{ccc} r_{post} & = & \exp\left[-\frac{\theta_{roll}^{2}+\theta_{pitch}^{2}}{\sigma_{\theta }^{2}}\right], \end{array} \end{array}$$ (19)

$$\begin{array}{c} \begin{array}{ccc} r_{cont} & = & 1-\frac{1}{4}\sum_{i=1}^{4}|c_{i}-{\bar{c}}_{i}|, \end{array} \end{array}$$ (20)

$$\begin{array}{c} \begin{array}{ccc} r_{clr} & = & \frac{1}{4}\sum_{i=1}^{4}(1-{\bar{c}}_{i})\min\left(\frac{z_{i}-z_{0,i}}{h_{t}+\epsilon_{z}},1\right), \end{array} \end{array}$$ (21)

$$\begin{array}{c} \begin{array}{ccc} r_{pow} & = & \sum_{k=1}^{8}|{\hat{\tau }}_{k,t}{\dot{q}}_{k,t}|, \end{array} \end{array}$$ (22)

$$\begin{array}{c} \begin{array}{ccc} r_{slip} & = & \frac{1}{4}\sum_{i=1}^{4}c_{i}\|{𝐯}_{i,xy}^{foot}{\|}_{2}, \end{array} \end{array}$$ (23)

$$\begin{array}{c} \begin{array}{ccc} r_{sm} & = & \|{𝐚}_{t}-{𝐚}_{t-1}{\|}_{2}^{2}. \end{array} \end{array}$$ (24)

Here $v_{x}$ is the measured forward body velocity, $v_{x}^{⋆}$ is the target velocity, $z_{i}$ is the foot height, ${𝐯}_{i,xy}^{foot}$ is the horizontal foot velocity, ${\hat{\tau }}_{k,t}$ is the estimated joint effort, and $\epsilon_{z}=10^{-4}$ avoids division by zero. We used $\sigma_{v}=0.15$ m/s and $\sigma_{\theta }=0.35$ rad. For the low-cost servo platform, direct torque sensing was unavailable; therefore, ${\hat{\tau }}_{k,t}$ was estimated from the calibrated servo command-tracking error and used consistently for both the reward penalty and the CoT estimate. The terminal reward $r_{term}=-1$ is applied when the robot falls, exceeds the safety tilt threshold, or terminates the traversal prematurely; otherwise $r_{term}=0$.

The coordination penalty is defined as


$$\begin{array}{c} r_{coord}=\frac{1}{4}\sum_{i=1}^{4}|c_{i}-{\bar{c}}_{i}|+\eta_{f}\;Var(\{\Delta f_{i}{\}}_{i=1}^{4})+\eta_{h}\;Var(\{\Delta h_{i}{\}}_{i=1}^{4}). \end{array}$$ (25)

This term penalizes disagreement between realized and desired contact schedules and discourages excessive divergence among the leg agents in their frequency and swing-height corrections. The reward weights were chosen by first normalizing each reward component to a comparable numerical range on nominal flat-ground rollouts and then increasing the velocity and posture weights until the controller tracked the commanded speed without excessive body attitude variation. The penalties on power, slip, smoothness, and coordination were kept smaller to regularize the learned behavior without overriding traversal success. In practice, each agent receives the same team reward, which promotes cooperation rather than competitive local behavior.

Training is conducted in Isaac Sim with 20 s episodes and domain randomization over terrain and robot parameters. During training, ground friction is randomized in [0.5,1.0], base mass is perturbed by ±10%, slope is randomized within $\pm 8^{\circ }$, and lateral push disturbances of 2–4 N are applied for 0.15 s at random times. A simple curriculum is used: obstacle height is gradually increased from 5 mm to 20 mm once the success rate over the previous evaluation window exceeds 85%. The multi-agent policy is evaluated every 20 ms, corresponding to the 50 Hz coordination layer.

### 2.6 Core advantages and low-cost platform integration

The low-cost merit of the proposed method is produced by the algorithmic decomposition rather than by the robot price alone. The CPG supplies a stable nominal gait using a small set of oscillator states; consequently, the learned policy solves only a bounded residual-correction problem. Parameter sharing allows all four leg agents to reuse the same actor, while CTDE removes the centralized critic from online execution. The controller also avoids an online rigid-body dynamics solver or receding-horizon optimizer: its runtime operations consist of fixed-dimensional CPG integration, planar inverse kinematics, shared-actor inference at 50 Hz, residual addition, and simple command projection at 200 Hz.

The sensing burden is similarly limited. Runtime observations are obtained from the IMU, joint states, and foot-contact signals already available on the platform. The method does not depend on an external motion-capture system, a terrain-reconstruction network, or direct joint-torque measurements. The monocular camera installed on the PuppyPi platform was not used by the reported controller. Table 2 summarizes the online implementation characteristics that support the low-cost claim.

**Table 2:** Online implementation characteristics of the proposed controller. Training-side resources such as the centralized critic, PPO optimizer, rollout buffers, and parallel simulation environments are excluded because they are not deployed on the robot.

Aspect	Proposed design	Low-cost implication
Learned model	One shared 23–256–128–4 actor for all four legs	39,560 parameters; approximately 155 KiB in 32-bit precision; 75% less actor storage than four independent copies
Online inference	Four actor evaluations at 50 Hz	Approximately $7.83\times 10^{6}$ multiply–accumulate operations per second; no centralized critic at runtime
Sensing	IMU, joint states, and foot-contact signals	No external motion capture, vision-based terrain reconstruction, or direct torque sensing
Low-level control	Hopf-CPG update, planar inverse kinematics, residual addition, clipping, rate limiting, and first-order filtering	Fixed-dimensional, non-iterative operations; no online MPC or full-body trajectory optimization
Hardware adaptation	Joint-limit projection, rate-limit projection, command smoothing, and neutral-pose reset	Protects inexpensive servos from saturation and command chattering without redesigning the learned policy

Actuation commands generated by the CPG and multi-agent coordination modules are mapped to the low-cost actuators through a hardware abstraction layer. The final command ${𝐪}_{t}^{cmd}$ is clipped to the joint range of each servo and then rate-limited before transmission to the motor controller. A first-order filter,


$$\begin{array}{c} {\tilde{𝐪}}_{t}=\rho {\tilde{𝐪}}_{t-1}+(1-\rho ){𝐪}_{t}^{cmd},\;\rho =0.8, \end{array}$$ (26)

is used to suppress command chattering. Safety logic also stops the gait and resets the robot to a neutral posture if sustained saturation or excessive tilt is detected. The hardware abstraction and bounded residual interface allow the same learned actor to be transferred between servo-based platforms by changing joint calibration, limits, and command scaling rather than redesigning the policy architecture.

### 2.7 Integrated control loop

The online implementation is executed as a two-rate closed loop. Let *k* denote the index of the 200-Hz low-level cycle. Because the policy layer operates at 50 Hz, the shared actor is evaluated when mod(k,4)=0. The most recently computed policy outputs are applied by zero-order hold during the remaining low-level cycles. The implementation proceeds as follows:

1. **Sensor acquisition and preprocessing:** Read the IMU orientation and angular velocity, joint angles and velocities, and foot-contact measurements at 200 Hz. Filter and temporally synchronize the measurements to suppress high-frequency sensing noise.
2. **State construction:** Construct the global state $s_{t}$, the four local observations $\{o_{i}{\}}_{i=1}^{4}$, and the shared coordination message $m_{t}$ from the filtered measurements, oscillator states, previous actions, and target forward speed.
3. **Multi-agent policy update:** If mod(k,4)=0, evaluate the shared actor independently for the LF, RF, LH, and RH leg agents to obtain the local actions defined in Eq. (11). Concatenate the local joint residuals according to Eq. (12).
4. **Gait-parameter aggregation:** Average the four frequency and swing-height proposals using Eq. (13), and clip the common gait parameters according to Eq. (14). The resulting $\Delta {𝐪}_{t}^{MA}$, $f_{t}$, and $h_{t}$ are held constant until the next 50-Hz policy update.
5. **CPG update:** Integrate the coupled oscillator dynamics in Eqs. (2)–(3) using the current contact feedback, IMU attitude feedback, and common gait parameters. The updated oscillator states determine the desired contact schedule and gait phase of each leg.
6. **Trajectory generation and inverse kinematics:** Map the oscillator states to desired Cartesian foot trajectories using Eq. (7), and calculate the nominal hip and knee targets ${𝐪}_{t}^{CPG}$ through inverse kinematics.
7. **Command fusion and hardware projection:** Combine the nominal and residual joint commands according to Eq. (1). Apply the joint-position limits and command-rate limits before passing the command to the hardware interface.
8. **Command filtering and actuation:** Apply the first-order command filter in Eq. (26), and transmit the filtered joint targets to the low-level servo controller.
9. **Safety supervision:** Continuously monitor body tilt, sustained joint saturation, and command-rate violations. If a safety condition is detected, stop the gait command and return the robot to its predefined neutral posture.

The resulting robot motion produces the measurements used in the next control cycle, thereby closing the sensing–control loop. This implementation preserves the interpretability and rhythmic stability of the 200-Hz CPG controller while allowing the 50-Hz leg-level policy to adapt the joint targets, step frequency, and swing height to terrain variations and asymmetric contact events without sacrificing whole-body gait coherence.

## 3 Experiments

### 3.1 Experimental setup

To evaluate the proposed framework, we conducted experiments in both simulation and real-world environments using a low-cost quadruped robot platform. The evaluation focuses on four representative terrain scenarios: *ramp*, *stairs*, *obstacles*, and *uneven terrain*. These scenarios were selected because they represent typical locomotion challenges for low-cost quadrupeds, including slope adaptation, discrete foothold placement, disturbance rejection, and robustness on continuously varying surfaces.

**Fig 2:**
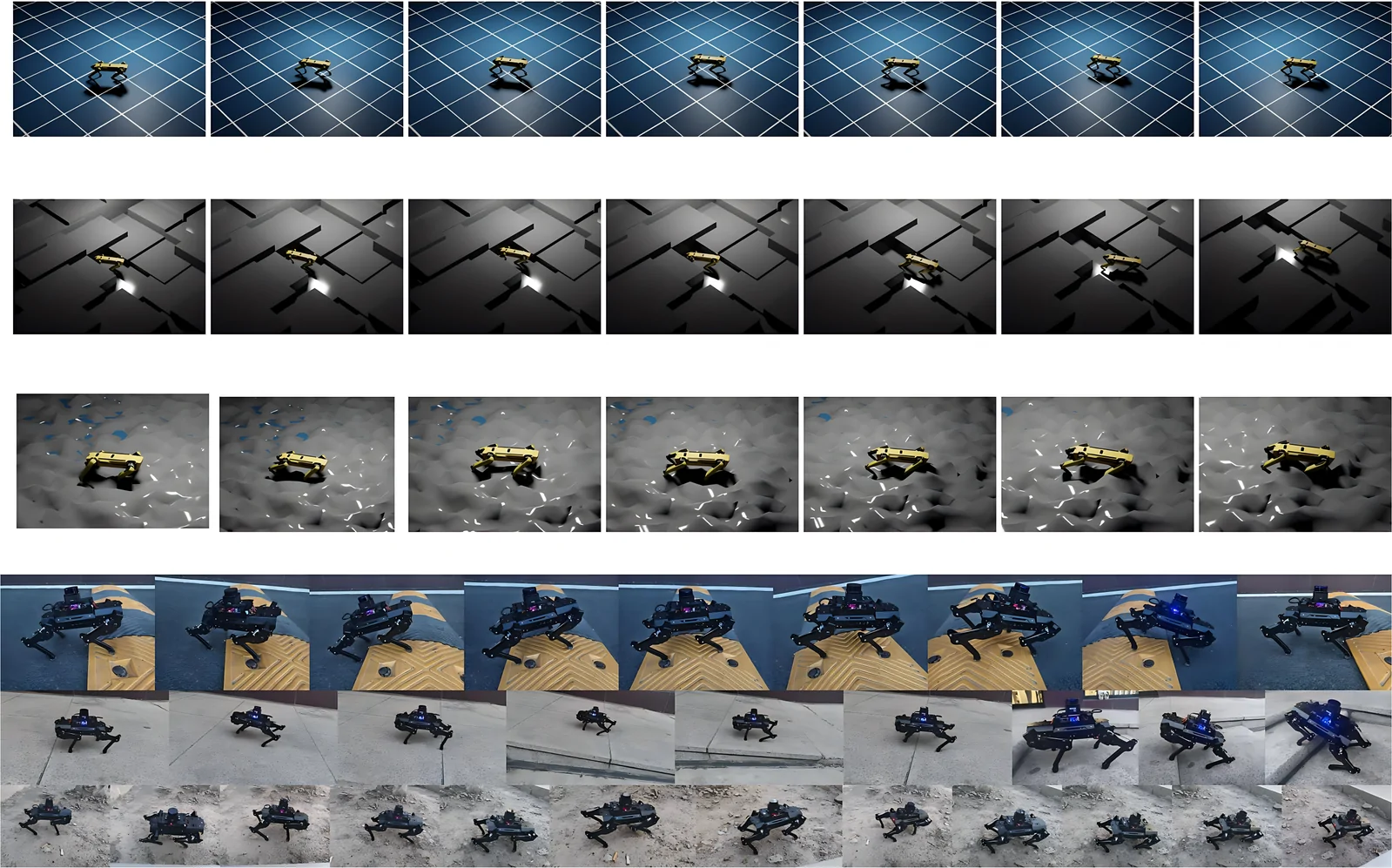
Overview of the experimental environments. The upper panel shows the simulation environments used for training and evaluation across ramp, stairs, obstacles, and uneven-terrain scenarios. The lower panel shows representative real-world experiments in the corresponding terrains.

Fig 2 presents representative snapshots of the simulation environments and corresponding real-world experiments. The same control architecture is deployed in both simulation and hardware experiments. The low-level CPG and servo loop runs at 200 Hz, while the high-level coordination policy runs at 50 Hz.

In all scenarios, the robot is commanded to move forward at a constant target speed. Each controller–terrain pair is evaluated over 20 independent trials. A trial is considered successful if the robot completes the assigned traversal without falling. A failure is declared when the robot loses balance, undergoes unrecoverable body collapse, or terminates locomotion prematurely.

**Physical hardware setup.** The physical platform used for experimental validation was the Hiwonder PuppyPi quadruped robot. The robot is a low-cost aluminum-alloy quadruped platform with 8 degrees of freedom, powered by a 7.4 V 2200 mAh LiPo battery and equipped with an onboard Raspberry Pi controller, a dedicated expansion board, and a monocular camera. Although the camera was physically present, camera images were not used by the reported controller. Online feedback was limited to IMU measurements, joint-angle feedback, and foot-contact signals; no external motion-capture system or direct torque sensors were required. This hardware configuration is suitable for evaluating whether the proposed controller can maintain robust locomotion under the sensing, computation, and actuation constraints typical of affordable quadruped platforms.

### 3.2 Compared controllers

We compare four controllers. All controllers use the same joint limits, command-rate limits, low-level servo interface, safety termination conditions, target speed, and evaluation terrains, so that the comparison isolates the controller architecture rather than hardware settings.

- **CPG-only**: this baseline uses Eqs. (2)–(7), the same inverse kinematics, and the same safety projection, but sets $\Delta {𝐪}_{t}^{MA}=0$, $\Delta f_{i}=0$, and $\Delta h_{i}=0$. It therefore tests the nominal oscillator and feedback controller without learned residual adaptation.
- **Multi-Agent-only**: this baseline keeps the leg-level CTDE policy and shared actor, but removes the CPG prior by replacing ${𝐪}_{t}^{CPG}$ with a neutral standing pose. The agents directly output bounded joint increments for each leg. This evaluates whether leg-level multi-agent coordination alone can generate effective locomotion without the structured oscillator prior.
- **RL-only**: this baseline uses a single centralized PPO actor that maps the global state to an 8-dimensional joint command correction. It uses the same reward and training randomization, but removes the leg-level agent decomposition, the shared coordination message, and the CPG gait prior. This evaluates the effect of replacing the proposed modular residual structure with a monolithic learning controller.
- **Integrated**: the proposed controller combines the CPG gait generator, the shared-parameter leg-level residual policy, frequency and swing-height aggregation, and the hardware-aware command projection.

### 3.3 Evaluation metrics and statistical analysis

To ensure consistency between the evaluation design and the reported results, the following metrics are used throughout the experiments:

- **Pitch Mean and Pitch Std**: mean and standard deviation of body pitch angle measured by the IMU;
- **Roll Mean and Roll Std**: mean and standard deviation of body roll angle measured by the IMU;
- **Success Rate**: percentage of successful trials for each controller–terrain pair;
- **Cost of Transport (CoT)**: a dimensionless energy-efficiency metric estimated from actuation effort during traversal.

The CoT is computed as


$$\begin{array}{c} CoT=\frac{\sum_{t=1}^{T}\sum_{k=1}^{8}|{\hat{\tau }}_{k,t}{\dot{q}}_{k,t}|\Delta t}{mgd}, \end{array}$$ (27)

where *m* is the robot mass, *g* is gravitational acceleration, *d* is the forward distance traveled during the trial, Δt is the control interval, and ${\hat{\tau }}_{k,t}$ is the estimated effort of joint *k*. Because the physical platform does not provide direct torque sensing, ${\hat{\tau }}_{k,t}$ is obtained from the calibrated servo command-tracking error. The same estimator is used for all controllers, making the CoT values comparable across methods even though they should be interpreted as effort-based estimates rather than direct electrical-energy measurements.

These metrics jointly characterize body-attitude stability, traversal robustness, and energy efficiency. Lower pitch/roll mean and standard deviation indicate better body stabilization, higher success rate indicates stronger terrain adaptability, and lower CoT indicates more efficient locomotion. For continuous stability metrics, we report means and standard deviations over repeated trials and use Welch’s *t*-tests for pairwise comparisons between the integrated controller and the best-performing baseline in each scenario. For success rate, Wilson 95% confidence intervals and Fisher’s exact tests are used when integer success/failure counts are available. Multiple pairwise tests are interpreted with Holm–Bonferroni correction. Statistical results are used to qualify the strength of the conclusions rather than to claim universal superiority across all operating conditions.

## 4 Results

### 4.1 Overall performance

**Fig 3:**
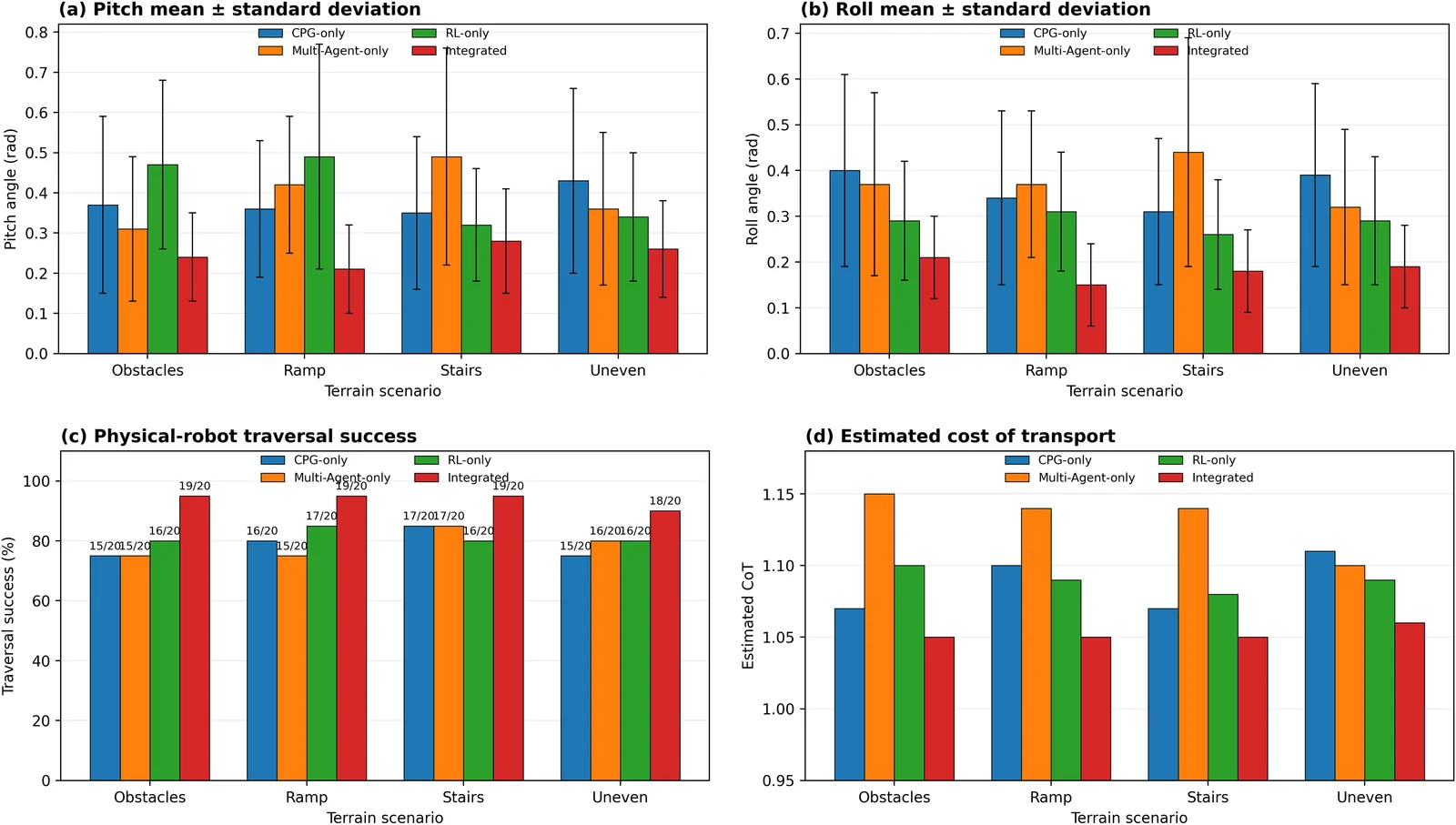
Physical-robot comparison of the four controllers across the obstacle, ramp, stair, and uneven-terrain scenarios. Panels (a) and (b) show the mean pitch and roll angles, respectively, with error bars representing one standard deviation over 20 independent trials. Panel (c) shows traversal success; the labels above the bars report the number of successful trials out of 20, and the bar heights show the corresponding percentages. Panel (d) reports the effort-based cost-of-transport estimate. Lower pitch, roll, and CoT values and higher success values indicate better performance.

**Fig 4:**
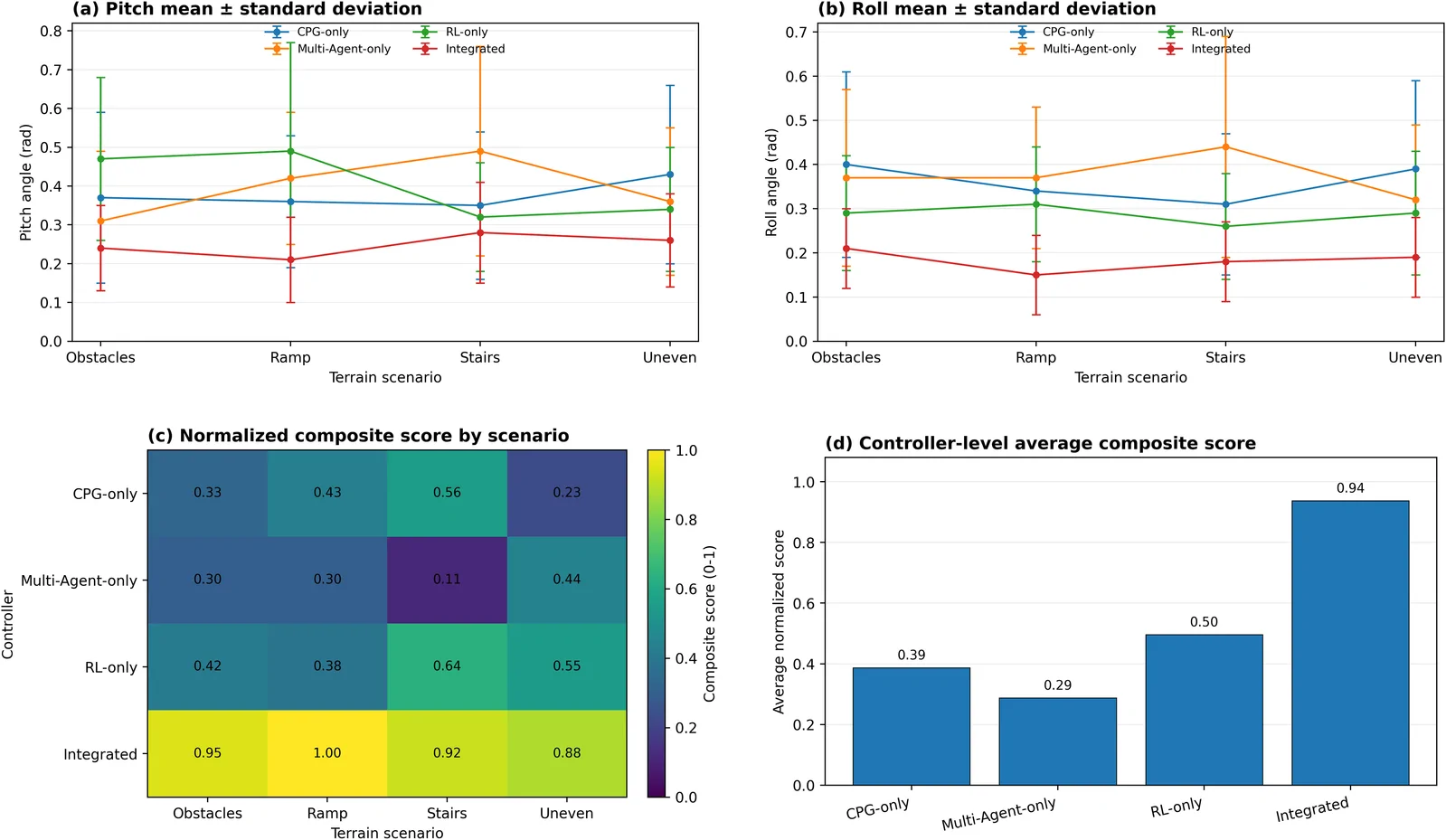
Additional physical-robot analysis synchronized with the revised 20-trial success results. Panels (a) and (b) show the pitch and roll means with one standard deviation over 20 independent trials. Panel (c) presents the scenario-level composite score obtained by globally min–max normalizing pitch mean, pitch standard deviation, roll mean, roll standard deviation, traversal success, and CoT, and then assigning equal weight to the six normalized metrics. Panel (d) reports the arithmetic mean of the four scenario scores for each controller. All scores range from 0 to 1, and higher values indicate a more favorable overall combination of stability, success, and estimated effort. The composite score is descriptive and should be interpreted together with the original metrics and statistical comparisons.

The physical-robot results are summarized in Figs 3–Fig 4, and Table 3. Fig 3 reports the continuous body-attitude metrics as the mean with one standard deviation over 20 independent trials, while traversal success is shown both as a percentage and as the underlying number of successful trials out of 20. Across the four scenarios, the integrated controller provides the most balanced performance, with comparatively small pitch and roll deviations, high traversal success, and low estimated CoT.

**Table 3:** Physical-robot performance of the four controllers across the obstacle, ramp, stair, and uneven-terrain scenarios. Each controller–terrain pair was evaluated over 20 independent trials. Continuous metrics are reported as the mean and standard deviation over the repeated trials. Traversal success is reported as the number of successful trials out of 20, with the corresponding percentage in parentheses.

Scenario	Controller	Pitch Mean	Pitch Std	Roll Mean	Roll Std	Success count (%)	CoT
Obstacles	CPG-only	0.37	0.22	0.40	0.21	15/20 (75.0)	1.07
	Multi-Agent-only	0.31	0.18	0.37	0.20	15/20 (75.0)	1.15
	RL-only	0.47	0.21	0.29	0.13	16/20 (80.0)	1.10
	Integrated	0.24	0.11	0.21	0.09	19/20 (95.0)	1.05
Ramp	CPG-only	0.36	0.17	0.34	0.19	16/20 (80.0)	1.10
	Multi-Agent-only	0.42	0.17	0.37	0.16	15/20 (75.0)	1.14
	RL-only	0.49	0.28	0.31	0.13	17/20 (85.0)	1.09
	Integrated	0.21	0.11	0.15	0.09	19/20 (95.0)	1.05
Stairs	CPG-only	0.35	0.19	0.31	0.16	17/20 (85.0)	1.07
	Multi-Agent-only	0.49	0.27	0.44	0.25	17/20 (85.0)	1.14
	RL-only	0.32	0.14	0.26	0.12	16/20 (80.0)	1.08
	Integrated	0.28	0.13	0.18	0.09	19/20 (95.0)	1.05
Uneven	CPG-only	0.43	0.23	0.39	0.20	15/20 (75.0)	1.11
	Multi-Agent-only	0.36	0.19	0.32	0.17	16/20 (80.0)	1.10
	RL-only	0.34	0.16	0.29	0.14	16/20 (80.0)	1.09
	Integrated	0.26	0.12	0.19	0.09	18/20 (90.0)	1.06

To provide a transparent descriptive summary across the six reported metrics, we compute a composite score using global min–max normalization over all 16 controller–terrain combinations. For the lower-is-better metrics (pitch mean, pitch standard deviation, roll mean, roll standard deviation, and CoT), the normalized score is


$$\begin{array}{c} z_{c,s,m}^{-}=\frac{x_{m}^{\max}-x_{c,s,m}}{x_{m}^{\max}-x_{m}^{\min}}, \end{array}$$

whereas traversal success is normalized as


$$\begin{array}{c} z_{c,s,succ}^{+}=\frac{x_{c,s,succ}-x_{succ}^{\min}}{x_{succ}^{\max}-x_{succ}^{\min}}. \end{array}$$

Here, $x_{c,s,m}$ denotes metric *m* for controller *c* in scenario *s*, and the minimum and maximum are calculated globally for each metric. The six normalized values are equally averaged to obtain the scenario-level composite score. The controller-level score is the arithmetic mean of its four scenario scores. This composite index is used only as a descriptive visualization and does not replace the individual metrics or the statistical tests.

The pitch and roll results indicate that the integrated controller reduces both the average body-angle deviation and the trial-to-trial variability in most tested cases. After recalculation using the corrected 20-trial traversal-success values, its normalized composite scores are 0.95, 1.00, 0.92, and 0.88 for the obstacle, ramp, stair, and uneven-terrain scenarios, respectively, with a controller-level average of 0.94. The corresponding average scores of CPG-only, Multi-Agent-only, and RL-only are 0.39, 0.29, and 0.50. These values provide a compact descriptive summary of the reported metrics; because the score uses global min–max normalization and equal metric weights, the individual measurements and statistical tests remain the primary basis for interpretation.

### 4.2 Statistical comparison

Table 4 reports Welch-test comparisons between the integrated controller and the best baseline for pitch and roll in each scenario, using the reported trial mean and standard deviation. The analysis shows that the roll-angle reduction is consistently favorable, while the pitch-angle reduction is statistically strong on the ramp scenario and more trend-like in the other scenarios. Therefore, the revised conclusions are phrased in terms of a favorable overall trade-off rather than uniform statistically significant improvement for every metric.

**Table 4:** Statistical comparison between the integrated controller and the best baseline for body-attitude metrics. Values are Welch-test *p*-values computed from the reported mean, standard deviation, and 20 repeated trials.

Scenario	Pitch baseline	Pitch *p*	Roll baseline	Roll *p*
Obstacles	Multi-Agent-only	0.148	RL-only	0.030
Ramp	CPG-only	0.002	RL-only	<0.001
Stairs	RL-only	0.355	RL-only	0.023
Uneven	RL-only	0.082	RL-only	0.011

### 4.3 Ablation interpretation

The three baselines also serve as component ablations of the integrated controller. CPG-only removes learned residual adaptation and the multi-agent policy; Multi-Agent-only removes the structured CPG prior; and RL-only removes both the leg-level decomposition and the oscillator prior in favor of a monolithic learned policy. The performance gap between these baselines and the integrated controller suggests that the best results are obtained when rhythmic priors, leg-level residual adaptation, and hardware-aware projection are used together. However, the present data do not fully isolate the coordination regularizer $r_{coord}$ from the rest of the multi-agent architecture. For this reason, we describe $r_{coord}$ as a stabilizing regularization term and avoid claiming that it is independently necessary unless an additional “Integrated without $r_{coord}$” experiment is added.

### 4.4 Scenario-wise results

In the obstacle scenario, the integrated controller achieves the best overall performance, with lower body-angle deviation and higher success rate than the baselines. This suggests stronger disturbance rejection when the robot encounters scattered local protrusions.

In the ramp scenario, the integrated controller also produces the lowest pitch and roll deviations and the highest success rate. This indicates better slope adaptation and more stable body posture during ascent and descent.

In the stair scenario, the integrated controller maintains the highest success rate together with low body-attitude variation. This result suggests improved foot clearance and more reliable inter-leg coordination during step negotiation.

In the uneven-terrain scenario, the same overall trend is preserved. The integrated controller again shows lower body-angle deviation, higher success rate, and lower CoT than the other controllers. This indicates that the framework remains effective not only for discrete terrain events, but also for continuously varying surfaces.

### 4.5 Controller comparison

The baseline controllers exhibit different strengths but also clear limitations. The CPG-only controller preserves regular rhythmic motion and relatively low energy consumption, but its adaptability is limited on more challenging terrain. The RL-only controller provides adaptive capacity, but its performance is less consistent across stability, success rate, and efficiency. The Multi-Agent-only controller improves coordination flexibility, but without the structured gait prior it does not achieve the same overall balance as the integrated framework.

By comparison, the integrated controller provides the most consistent trade-off among the reported metrics in the tested scenarios, which is particularly important for practical deployment on low-cost quadruped platforms. The statistical analysis indicates that some improvements, especially roll stabilization, are stronger than others; therefore, the method should be viewed as improving the overall balance of stability, success rate, and effort rather than dominating every metric in every terrain.

### 4.6 Sim-to-real consistency

The simulation and real-world results show a consistent overall trend. In both domains, the integrated controller provides the most favorable trade-off among body-attitude stability, traversal success rate, and energy efficiency. This consistency suggests that the proposed combination of CPG-based gait generation, leg-level multi-agent coordination, and hardware-aware command projection transfers reasonably well from Isaac Sim to the physical low-cost platform.

At the same time, some discrepancy between simulation and hardware is expected. The physical robot is affected by actuator backlash, battery-voltage variation, contact uncertainty, and sensing noise that can only be approximated in simulation. The preserved controller ranking suggests that the proposed framework transfers reasonably well to the PuppyPi platform, but broader deployment claims should be verified on additional robots, speeds, and terrain geometries.

## 5 Discussion and future work

The core advantage of the proposed framework is the division of the locomotion problem according to computational role. The CPG handles nominal rhythmic generation, the compact shared actor supplies only bounded residual and gait-parameter corrections, and the hardware interface enforces actuator-safe commands. This division reduces the scope of the learned problem and avoids using a large monolithic policy to reproduce the complete gait. Parameter sharing, local observations, and CTDE further reduce online storage and inference requirements because only one actor is deployed and the centralized critic remains offline.

The low-cost claim therefore has three distinct meanings. First, the computational cost is limited by a 39,560-parameter shared actor evaluated at 50 Hz together with fixed-complexity CPG and inverse-kinematics operations. Second, the sensing cost is limited because the controller uses IMU, joint, and contact feedback without external motion capture, direct torque sensing, or vision-based terrain reconstruction. Third, the integration cost is reduced by residual commands, joint/rate projection, filtering, and hardware abstraction, which accommodate inexpensive servo limitations without embedding an iterative model-based optimizer in the online loop.

A key observation is that the integrated controller improves the overall balance between stability and CoT in the tested scenarios. This suggests that the performance gain is not only caused by larger corrective actions, but also by organizing the learned corrections around a structured gait prior and bounded hardware-aware commands. Within the tested range of terrains and speeds, the results support the view that rhythmic priors, modular coordination, and hardware-constrained control can improve practical locomotion on affordable hardware.

The low-cost characterization does not imply that the offline training process is inexpensive. Training still uses parallel simulation and a large number of simulator steps; the claimed savings apply to the deployed controller and its sensing/hardware requirements. This study also has several limitations. First, the complexity values reported in Table 2 are analytical model-size and operation-count estimates rather than processor-specific measurements of latency, memory bandwidth, or electrical power. Second, the current evaluation is mainly based on aggregate metrics rather than detailed per-trial trajectory analysis. Third, although the baselines isolate the CPG prior, multi-agent decomposition, and monolithic RL alternative, a dedicated ablation that disables only the coordination regularizer $r_{coord}$ should be added to quantify its independent effect. Finally, the study focuses on forward locomotion and does not yet examine turning, dynamic gait transitions, or vision-guided foothold planning.

Future work will benchmark inference latency, memory use, and power consumption on multiple embedded processors; strengthen the sim-to-real analysis; report larger-sample statistical tests with raw trial data; add a dedicated coordination-regularizer ablation; and explore richer multi-agent communication and exteroceptive perception for more complex locomotion tasks.
